# Flexural Strength and Stress Analysis of CAD-CAM Milled Glass Fiber Post and Core

**DOI:** 10.1155/ijod/6298753

**Published:** 2025-04-26

**Authors:** Sergio Eduardo Ramos dos Santos, Klíssia Romero Felizardo, Ricardo Danil Guiraldo, Sandrine Bittencourt Berger, Murilo Baena Lopes

**Affiliations:** ^1^Department of Dentistry, Universidade Norte do Paraná, Londrina, PR, Brazil; ^2^Department of Dentistry, Universidade Paranaense, Umuarama, PR, Brazil; ^3^Department of Dentistry, Anhanguera-Uniderp University, Campo Grande, Mississippi, Brazil

**Keywords:** CAD-CAM, dental posts, elasticity imaging techniques, flexural strength (FS)

## Abstract

Intraradicular posts have the potential to induce mechanical stresses within the root structure during masticatory activities, hence contributing to fatigue and ultimately resulting in the fracture of the remaining dental structure. The objective of this work was to compare the stress pattern generated on the root by a computer-aided design and computer-aided manufacturing (CAD-CAM) milled fiberglass post under occlusal load in a photoelastic simulation and to compare its flexural strength (FS) to other types of posts. A total of 15 simulated roots, produced from photoelastic resins, were created using human canine teeth. These specimens were then separated into three distinct groups: Fiber post conventional, which consisted of fiberglass posts; fiber post customizable, which involved prefabricated fiberglass posts anatomized with composite resin; and fiber post CAD-CAM, which utilized CAD-CAM milled fiberglass posts. Following the application of Relyx ARC cement, the posts underwent photoelastic testing using a polariscope and FS testing using a universal testing machine. The data were submitted to analysis of variance (ANOVA) and Tukey's test at 5% significance level. It was found that the stress was significantly greater (*p* < 0.05) for the customizable post (246.5 MPa ± 218.7) in comparison to both the conventional (135.8 MPa ± 99.3) and CAD-CAM (136.5 MPa ± 68.4) posts; the latter group exhibited the lowest flexural values (50.8 MPa ± 7.9), which were significantly different from both the conventional group (123.0 MPa ± 26.9) and the customized group (230.3 MPa ± 18.9), which also differed from each other. The modulus of elasticity showed statistical differences (*p*  < 0.05) among the three different methods: CAD-CAM (0.50 GPa ± 0.06), conventional (1.75 GPa ± 0.13), and custom (3.46 GPa ± 0.19). The FS and elasticity modulus of customized posts were significantly higher than those of CAD-CAM, that exhibited the lowest values. Intermediate values were demonstrated by conventional posts. In comparison, to the customized post, the stress study revealed that conventional and CAD-CAM posts had a lower stress in the apical area and a lower general root stress value; but the cervical stress from CAD-CAM posts were practically twice of conventional and customized posts.

## 1. Introduction

The use of intraradicular posts is indicated after endodontic treatment of teeth that have little remaining crown to allow the development of the form of resistance and retention of the core and crown [1–5]. Due to the considerable increase in esthetic demand, some posts have been introduced to the market, such as fiberglass, quartz, and ceramic posts [5–7]. These materials have optical properties similar to dentin and therefore allow for excellent aesthetic results with all-ceramic restorations, as well as preventing darkening of the root and gingiva from corrosion products [8]. Subsequently, the customizable post technique was introduced, where a fiberglass post is molded with composite resin in the canal to be cemented [9, 10].

An evolution of this technique is the total customization of the fiber post, which is made in the shape of the canal using computer-aided design (CAD) and computer-aided manufacturing (CAM) techniques. The entire post is made of a single fiber-based material, eliminating the fiber-resin interface, and possibly improving its mechanical properties. With the introduction of CAD-CAM technologies, a more anatomic glass fiber post with an elastic modulus similar to dentin and the ability to absorb masticatory stresses became available [11, 12]. Nonetheless, the performance of CAD-CAM FRC systems for endodontically treated tooth restoration has received little attention [13].

The elastic modulus is a significant predictor of the performance of intraradicular materials. High rigidity materials tend to concentrate stress on the remaining tooth structure during restoration, thereby increasing the risk of fracture. FRC posts exhibit enhanced aesthetic properties and an elastic modulus comparable to that of dentin [14], resulting in a reduced incidence of irreparable root fractures and aesthetic issues relative to metallic posts [15, 16].The use of materials with an elastic modulus comparable to that of dentin results in a more uniform distribution of stresses [17, 18]. Intraradicular posts with lower elastic modulus cause deformation in the tooth, cement, and post at the weakest point, which is the luting cement–dentin interface, during occlusal function. This results in the loss of marginal seal, fracture of the internal root or the root itself, and/or loss of retention [19]. In rigid posts, forces are concentrated at the root, resulting in fatigue failure and the potential for catastrophic or irreparable fractures [19].

The advantage of fiberglass posts is that their modulus of elasticity is similar to that of dentin, meaning there is less concentration of masticatory forces at the apex of the tooth, as opposed to metal posts, which can generate areas of concentrated tension, resulting in cracks and fractures in the tooth structure [20]. They also require less wear on the root canal, resulting in a larger root remnant [20]. However, they are more susceptible to method, necessitating more knowledge, and preparation from the dental surgeon, particularly during the cementation procedure and coronal restoration.

Anatomical posts are recommended for the rehabilitation of anterior teeth with wider canals and a large amount of afflicted dentin tissue [21]. In this approach, in addition to the fiberglass post, composite resin is used to fill the canal in order to reduce the gap that would otherwise be filled by resin cement, resulting in biomechanical behavior as a replacement for the missing dentin structure [22]. Anatomical posts are extremely effective in the treatment of fragile roots because they improve both aesthetics and masticatory function [23].

CAD-CAM posts can be utilized when there is no remaining crown and can support large prostheses. The key advantage over the old procedure is the speed with which the restoration is completed; nevertheless, clinical investigations are required to determine whether this method can produce crowns that are durable enough to persist for a long time [24, 25]. CAD-CAM technology includes advantages such as digital impressions and models as well as the usage of virtual articulators. However, the cost of implementing this technology remains significant, and highly trained individuals are required [26].

The stresses generated by the posts are the result of the masticatory forces imposed on the dental structures [27]. However, the stress is passed to the less rigid substrate (dentin) and can cause failure, which is why posts should have an elasticity modulus similar to dentin [2, 28]. Although some authors have analyzed the stress that occurs when using posts using finite element analysis and elasticity theory, the properties of biological structures are only approximate, the materials are considered homogeneous, isotropic and with a linear modulus of elasticity [7, 29–31]. Therefore, it is advisable to complement the results with clinical and laboratory tests. The photoelasticity method seems to be particularly acceptable for this purpose [32].

The clinical success of fiber posts is determined by the tooth shape, the position of the tooth in the arch, the adaptation to the root, the cementation technique, the remaining coronal structure, the ferrule presence, and adequate adhesive bond [33, 34]. There is a limited amount of available information regarding the CAD-CAM materials utilized in the manufacturing process of glass fiber posts. Consequently, it is necessary to assess the performance and characteristics of CAD-CAM technology [12, 35], because intraradicular posts exhibit varying biomechanical behavior. The goal of this work was to compare the stress pattern generated on the root by a CAD-CAM milled fiberglass post under occlusal load in a photoelastic simulation and to compare its flexural strength (FS) to other types of posts. The first hypothesis to be tested is that the CAD-CAM post distributes occlusal forces more evenly in the root structure. The second hypothesis is that the CAD-CAM post is more resistant to the FS test.

## 2. Materials and Methods

This research was submitted to the University Pitágoras-Unopar ethics committee, registered under number CCAE 30582820.3.0000.0108, and approved (report number 4.023.718) before being carried out. The research employed a canine tooth that had been surgically removed due to periodontal concerns from a participant who willingly participated in the study and provided informed consent by signing a consent form, which includes his consent to publish.

The sample calculation was based on a pilot study. For the photoelastic test, the maximum difference and the assumed standard deviation (SD) were calculated by region, respectively: apical - 280.00 MPa, 12.45, middle - 59.4 MPa, 25.78, and cervical - 38.4 MPa, 8.9. The results showed the need for three samples for the apical and cervical regions, whereas five samples for the middle region. Therefore, it was decided to use five photoelastic samples for each group. In the bending test, using a maximum difference of 195.72 MPa and an assumed SD of 59.35, the number of samples required was four. We opted to standardize all tests at five samples for each group.

### 2.1. Photoelastic Analysis

A photoelastic model was made from a selected human canine tooth with a length of 27 mm, with no external or internal changes to the root canal anatomy. The canine had its crown removed with a silicon carbide disk (EF002, American burs, Palhoça - SC, Brazil) 1 mm above the enamel/cementum junction. The end of the tooth remnant was prepared in the shape of a chamfer with a rounded edge truncated conical diamond tip (number 4137, KG Sorensen, São Paulo, SP, Brazil) at high speed. The manufacturer's recommendation for use with a #3 fiberglass post (Reforpost, Angelus, Londrina, PR, Brazil) was to prepare the root canal with parallel, slightly divergent walls using the largo bur sequence #2 to #5. The length of the root preparation was calculated by measuring the remaining tooth length and subtracting 4 mm from the apex.

**Figure 1:**
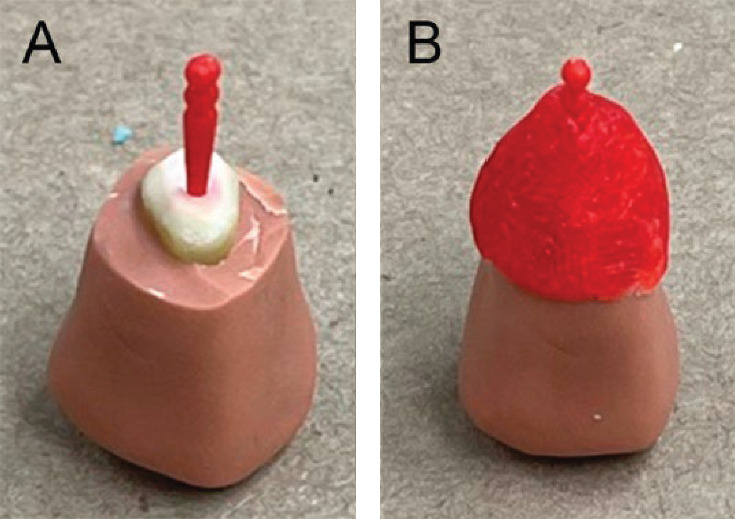
(A) Mold of the root using heavy body addition silicone and (B) mold of the coronal preparation using an acrylic resin tray and light body addition silicone.

Fifteen replicas were made using addition silicone (Aquasil, Dentsply, Rio de Janeiro, RJ, Brazil) and a polycarbonate post (Pin-Jet, Angelus, Londrina, PR, Brazil). The roots were first molded with heavy body silicone (Figure 1A) and then the coronal preparation with light body using an acrylic tray with internal relief (Figure 1B) [9].

**Figure 2:**
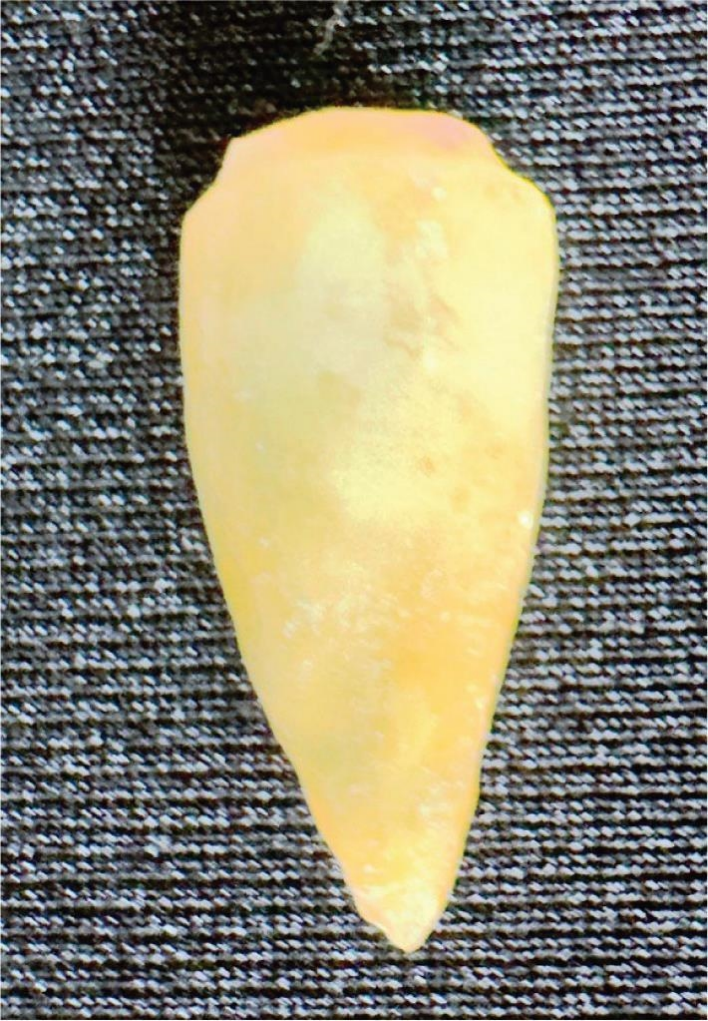
Photoelastic replica of the tooth.

To make the photoelastic model, PL-3 epoxy resin (Vishay Measurements Group, USA) was used, handled according to the manufacturer's instructions. The resin was poured into the heavy body addition silicone mold and the acrylic resin tray was placed on the heavy body silicone mold. It was kept in place for 48 h to obtain the photoelastic model (Figure 2).

All models were examined, and those with visible modifications or bubbles were rejected and remade. To achieve micromechanical retention, the inner walls of the photoelastic model canal were sandblasted with 50 m alumina particles. The specimens were protected with a silicone matrix, which left only the lumen of the canal exposed and the aluminum jet was then applied. It was then followed by finishing with 1200-grit sandpaper (3 M). The 15 roots simulated in photoelastic resin were randomly divided into 3 groups of 5 samples each: FP-Glass fiber post (Reforpost, Angelus, Londrina, PR, Brazil), FPC-prefabricated glass fiber posts (Reforpost, Angelus, Londrina, PR, Brazil) anatomized in composite resin (Z-350, 3M Espe, Saint Paul, MN, USA) and FCAD - CAD-CAM glass fiber post (Experimental, Angelus, Londrina, PR, Brazil).

**Figure 3:**
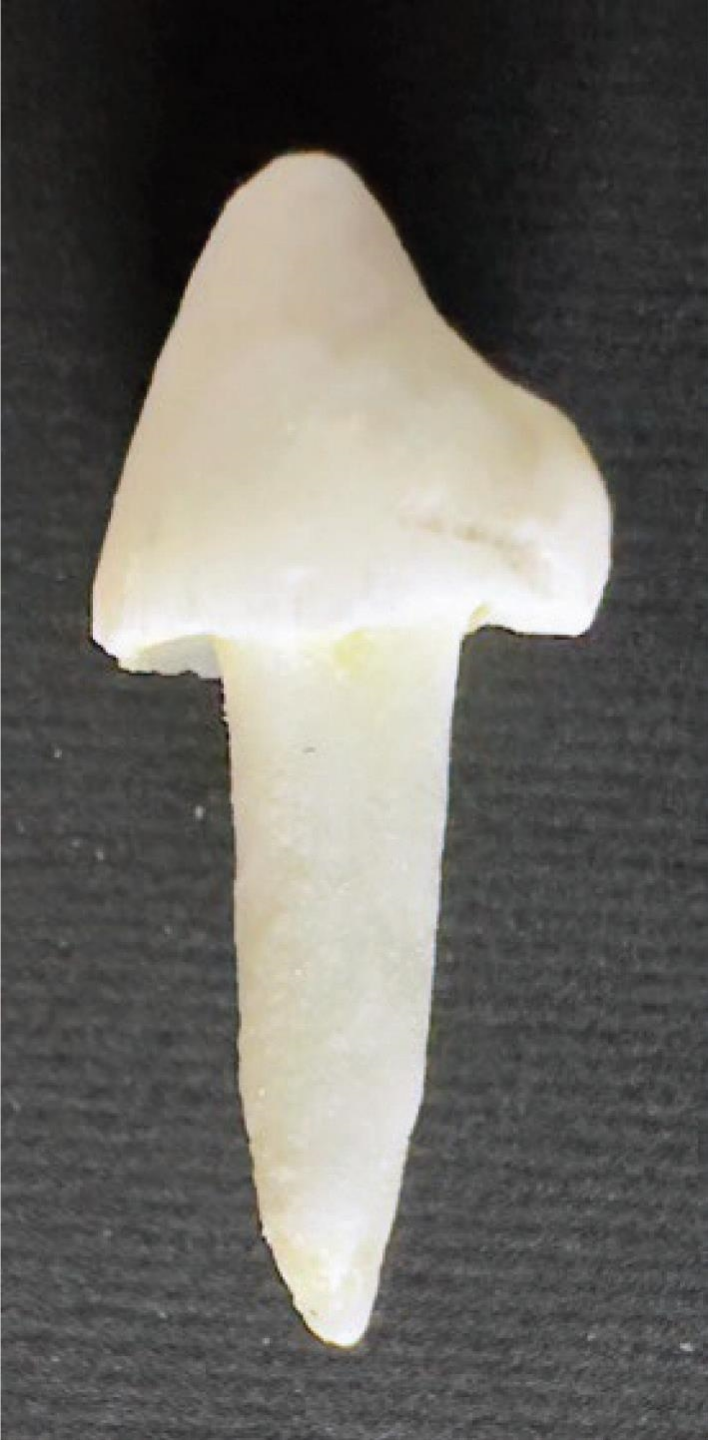
Fiber glass milled.

In FP group, a layer of silane (Angelus, Londrina, PR, Brazil) was applied to the post, followed by the application of adhesive (Adper Scotchbond Multipurpose, 3M ESPE, St Paul, MN, USA) to the canal and post, and photoactivated for 20 s. Using a Snap-Fit Syringe (Centrix Inc, Shelton, CT, USA), the resin cement RelyX ARC (3M ESPE, St Paul, MN, USA) was activated and placed within the photoelastic canal. The fiberglass post was inserted into the canal and polymerized for 40 s on each face (occlusal, mesial, buccal, distal and palatal). The EVA matrix was pressed into a CAD-CAM core and composite resin cores in Z350 (Filtek Z350, 3M ESPE, Irvine, CA, USA) were made over the posts using this EVA matrix (Figure 3). The resin was photoactivated (Grand VALO, Ultradent, Utah, USA), and the light intensity was measured using a radiometer for each group restored to guarantee that the power was greater than 1,000 mW/cm^2^.

In the FPC Group, a water-soluble gel (KY Gel, Johnson & Johnson) was used to isolate the canal of the photoelastic model. After silanizing the post, the composite resin (Filtek Z350, 3M ESPE, Irvine, CA, USA) was placed around it and the set was taken into the canal and photoactivated for 20 s. The composite post-resin set was removed from the canal and photoactivated for a further 40 s. After applying the adhesive (Adper Scotchbond Multipurpose) to the canal, the set was luted with a double activation resin cement (RelyX ARC). Composite resin cores were made in Filtek Z350 as described in FP group.

In the FCAD group, a fully modeled fiberglass post (FIbercad, Angelus, Londrina, PR, Brazil) was milled in a horizontal direction on a Ceramill Mind milling machine with a set scanner (Amann Girrbach AG, Austria). The root canal and coronal preparation were molded using addition silicone (Aquasil, Dentsply) and transferred to the CAD-CAM system, where a fiberglass replica was generated (Figure 3). The set was cemented with dual cure resin cement (RelyX ARC, 3M ESPE, St Paul, MN, USA). The cores milled served as a guide for making the cores for the other groups.

The specimens were kept in humidity at 37°C for 24 h. Around the intraradicular post, three sections were investigated: the cervical third, the middle third, and the apical third, each measuring 7 mm.

**Figure 4:**
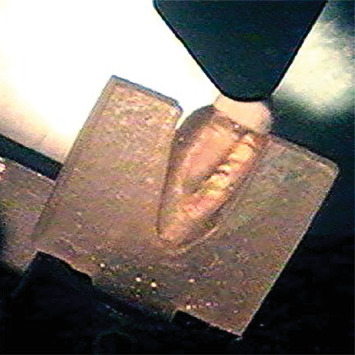
Specimen positioned and analyzed in a polariscope.

**Figure 5:**
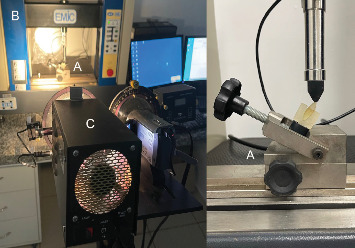
(A) Specimen positioned in an acrylic device with an axial force applied, (B) universal testing machine, and (C) polariscope.

A customized acrylic resin device was constructed and mounted to a stainless steel base to duplicate the axial position and observe the complete mesial and distal faces (Figure 4). The stresses were applied (Figure 5A) by loading through a load applied 2 mm from the incisal edge in the palatal region of the tooth by a Universal Testing Machine (EMIC DL 2000, Instron Brasil Equipamentos Científicos LTDA, São José dos Pinhais, PR, Brazil) (Figure 5B) were evaluated by photoelastic analysis on a polariscope (Photostress, Vishay Measurement Group USA) (Figure 5C). The data was obtained in MPa and submitted to the Kolgomorov–Smirnov normality test. As the data was found to be normal, the mean and SD were used to present the results using a parametric test. Data was submitted to ANOVA and Tukey's test at a 5% significance level.

### 2.2. FS and Modulus of Elasticity

The materials' FS was assessed using a three-point bending test adapted from ISO 4,049 [36].. Fifteen specimens in the shape of the root canal were obtained for the FV, FVC, and FVCC groups, according to the manufacturing technique described. The specimens were kept in distilled water at 37°C for 24 h. Each specimen's dimensions were measured with an accuracy of 0.01 mm using a digital caliper (Mitutoyo, Tokyo, Japan). The three-point bending test was performed at 0.5 mm/min on a mechanical testing machine (DL2000, EMIC). Each sample was placed on a device that had a 5 mm gap between two metal supports. The FS was computed as FS=3*P*_*f*_*L*/2*WH*^2^, where *P*_f_ was the maximum load required to fracture the specimen (*N*), *L* was the distance between the supports (5 mm), *W* was the specimen's width (mm), and *H* was its thickness (mm).

The bending test was monitored using software on a computer connected to the mechanical testing machine, which automatically generated a ‘stress strain' graph during the test. The modulus of elasticity (E) for each specimen was calculated using the formula: E=(*∆F*/*∆*y) x (*L*^3^/4*WH*^3^), where: *∆*F is the change in force per unit change in deflection of the center of the specimen (*∆*y), *L* is the distance between the supports (5 mm), *W* is the width of the specimen (mm), and *H* is the thickness (mm) [37]. At a 5% significance level, the data was submitted to the Kolgomorov–Smirnov normality test. As the data was found to be normal, the mean and SD were used to present the results using a parametric test. Data was submitted to ANOVA, and Tukey's test.

## 3. Results

### 3.1. Photoelastic Analysis

The photoelasticity analysis revealed that the CAD-CAM posts distributed forces similarly to the conventional posts (Table 1). The customized posts exhibited higher apical stresses than the other types (Table 2).

### 3.2. FS and Modulus of Elasticity

According to the findings of the bending tests, the CAD-CAM posts have lower bending strength and modulus of elasticity, followed by the customized and conventional posts (Table 3).

## 4. Discussion

CAD-CAM have become increasingly popular in dentistry. This method can be applied to inlays, onlays, veneers, crowns, fixed partial dentures, implant abutments, and even fullmouth reconstruction in the laboratory and in the dental clinic [38]. The utilization of scanning techniques on root canals or models presents the opportunity to create tailored structures that closely correspond to the dimensions and contours of the root canal system. These structures can be affixed with a thin coating of cement, resulting in enhanced adaptability [39, 40]. The use of CAD-CAM milled fiberglass blocks produces a monolithic core that eliminates the usage of different materials and the presence of multiple interfaces in the cemented structure [41, 42]. Although clinicians may prefer metal posts over prefabricated FRC posts for restoring severely damaged anterior teeth, prefabricated and CAD-CAM FRC post systems have more favorable failure modes and higher survival rates for missions that require anterior masticatory forces [43].

The first hypothesis was accepted as the stress analysis revealed that conventional and CAD-CAM posts had a lower tension (Table 1) and a more evenly distribution of forces throughout the root region (Table 2). The stress values of the CAD-CAM group in the apical and middle regions were statistically similar to those of the conventional post, but the cervical region showed greater stress. This greater stress in the cervical region may be associated with greater flexibility, which led to a greater similarity of values between the different regions of the post. In addition, the lower SD for the CAD-CAM group shows greater homogeneity between the specimens. This reduces the number of root fractures, particularly catastrophic failures in the apical and middle areas, which result in the loss of the dental element. The fibers have high tensile strength [44] and give an elastic matrix rigidity and resistance. Polymerization contraction is larger in the case of the customized post and the standard post, where a larger amount of cement is employed [19]. When the stresses were evaluated together, the customizable post showed a higher stress. This is most likely because the CAD-CAM post enters the root canal tightly, as it has not been subjected to the wear process to compensate the space needed for the cementing agent, which happens in the manufacturing process of the metal post when subjected to sandblasting. As a result, in addition to the higher stress, the post is likely to have some degree of maladaptation.

The second hypothesis were rejected as the higher FS was shown by the customized post, followed by the traditional post, and finally by the CAD-CAM post as it had a lower modulus of elasticity than the others. The tension generated in the roots is closely related to this feature. As observed in the CAD-CAM group, a lower modulus of elasticity is more conducive to stress distribution. Furthermore, when compared to prefabricated and customized fiberglass posts, the construction of a monolithic system has fewer voids and gaps, with equivalent push–out bond strength values [33].

When relating one test to another, the CAD-CAM post exhibited higher flexibility compared to the other groups, resulting in reduced stress in the cervical region. Nevertheless, the elevated stress in the apical region relative to the cervical region suggests that even while there is increased flexibility, force is still being transmitted to the apex. However, the apical tension exhibited a reduced intensity compared to the other groups.

The milling procedure weakens the fiberglass posts [21]. This could be due to fiber wear, which creates ends where the fibers can develop openings. In contrast, investigations on milled and prefabricated cemented posts reported comparable fracture test results [13, 40, 42]. The CAD-CAM group had lower fracture resistance, which could be countered by lower stress generation. The CAD-CAM group's continuous bidirectionally oriented long fibers are orthotropic, resulting in multidirectional reinforcement but at the expense of decreased strength in any one direction [45]. Although reinforcement is advantageous, an increased concentration of glass fibers in the polymeric matrix of fiber posts can lead to a greater occurrence of interfacial defects. The CAD-CAM post is more sensitive to this since it contains more fibers in its total volume because it is totally constructed of this material. In addition to fiber, the others have composite resin in the case of customizable posts and a larger amount of resin cement in the case of standard posts. This, in turn, may have an impact on the strength of the system, as effective interfacial bonding is crucial for efficient load transfer from the polymer matrix to the reinforcing fibers [13, 43, 46]. CAD-CAM glass fiber posts have an elastic modulus similar to dentin [14], which optimizes the post and restoration system's strength. Furthermore, they adapt better to the root canal, resulting in less cement thickness at the bond interface and better stress distribution [14]. The manufacturing procedure, which is directly related to fiber orientation, post adaptation, and stress dissipation at the post-cement-dentin interface, can also affect the mechanical behavior of glass fiber posts [33, 41].

The fact that the specimens used for the bending test and determining the modulus of elasticity had the shape of the actual post utilized in the clinical situation is a limitation of this investigation. The posts' shape, which deviated from ISO 4049 recommendations, had an impact on them as a result. In addition, a ferrule is commonly advised as an optimal design for the restoration of a tooth without pulp, in conjunction with a post and core. Another limitation was the use of a single brand of conventional and CAD-CAM post. As a result, if different brands are used, the findings may differ, although the overall trend is likely to be similar. Nevertheless, it is not uncommon to meet exceptional circumstances in routine clinical practice. of patients having very limited remaining tooth structure [47]. However, during a Finite Element Analysis, it was determined that both the cast post and fiber post exhibited comparable qualitative and quantitative stress on the dentin when subjected to a standard functional biting load. This suggests that both types of posts are suitable for withstanding functional loads [48].

Clinically, decreased strain in the apical region is beneficial since it reduces the likelihood of a catastrophic fracture, resulting in tooth loss. The interlacing layers of fiberglass and resin (55% wt glass fibers) inserted in the polymer matrix (45% epoxy resin) of the CAD-CAM posts produce an elastic modulus similar to that of dentin [49]. The inclusion of glass or silica fibers into these posts has resulted in great esthetics, albeit flaws have been recorded [50]. An additional benefit of utilizing CAD-CAM FRC posts is the growing utilization of technology instruments, such as scanners, and virtual tools in the regular practice of clinicians. This enables the CAD designing and milling of personalized FRC posts that eliminate the need for resin core buildup [43].. The primary benefit of the CAD-CAM technique, in contrast to traditional methods, is the rapidity of restoration production. Nevertheless, clinical studies are required to evaluate the long-term efficacy of this method in producing durable crowns [24, 25]. The study serves as a prediction of performance, which is crucial to know given that this material has only recently been used clinically.

## 5. Conclusions

The FS and elasticity modulus of customized posts were significantly higher than those of CAD-CAM, which exhibited the lowest values. Intermediate values were demonstrated by conventional posts.

Comparatively to the customized post, the stress study revealed that conventional and CAD-CAM posts had a lower stress in the apical area and a lower general root stress value. But the cervical pressures from CAD-CAM posts were practically twice those of conventional and customized posts.

## Figures and Tables

**Table 1:** Tension (MPa) according to post type independent of the stress location.

Conventional	135.8 MPa ± 99.3b

Customized	246.5 MPa ± 218.7a

CAD-CAM	136.5 MPa ± 68.4b

*Note:* Different letters indicate statistical difference using Tukey's test at 5% significance level.

**Table 2:** Stress (MPa) considering post type and force location.

Post type/force location	Cervical	Middle	Apical
Conventional	23.2 MPa ± 6.7Aa	140.8 MPa ± 35.3Ab	243.6 MPa ± 53.0Ac
Customized	29.4 MPa ± 3.85Aa	183.8 MPa ± 62.1Ab	526.2 MPa ± 43.4Bc
CAD-CAM	55.8 MPa ± 10.8Ba	139.6 MPa ± 6.9Ab	214.2 MPa ± 22.2Ac

*Note:* Different uppercase letters in the column and lowercase letters in the row indicate statistical differences using Tukey's test at 5% significance level.

**Table 3:** Flexural Strength (MPa) and Modulus of Elasticity (GPa).

Post type/properties	Flexural Strength	Modulus of elasticity
Conventional	123.0 MPa ± 26.9b	1.75 GPa ± 0.13b
Customized	230.3 MPa ± 18.9a	3.46 GPa ± 0.19a
CAD-CAM	50.8 MPa ± 7.9c	0.50 GPa ± 0.06c

*Note:* Different letters indicate statistical differences using the Tukey test at 5% significance level.

## Data Availability

The laboratorial data used to support the findings of this study are available from the corresponding author upon request.

## References

[1] Assif D., Gorfil C. (1994). Biomechanical Considerations in Restoring Endodontically Treated Teeth. *The Journal of Prosthetic Dentistry*.

[2] Bateman G., Ricketts D. N., Saunders W. P. (2003). Fibre-Based Post Systems: A Review. *British Dental Journal*.

[3] Dietschi D., Duc O., Krejci I., Sadan A. (2007). Biomechanical Considerations for the Restoration of Endodontically Treated Teeth: A Systematic Review of the Literature--Part 1. Composition and Micro- and Macrostructure Alterations. *Quintessence International*.

[4] Morgano S. M., Brackett S. E. (1999). Foundation Restorations in Fixed Prosthodontics: Current Knowledge and Future Needs. *The Journal of Prosthetic Dentistry*.

[5] Schwartz R. S., Robbins J. W. (2004). Post Placement and Restoration of Endodontically Treated Teeth: A Literature Review. *Journal of Endodontics*.

[6] Qualtrough A. J., Mannocci F. (2003). Tooth-Colored Post Systems: A Review, Oper Dent.

[7] Toksavul S., Zor M., Toman M., Gungor M. A., Nergiz I., Artunc C. (2006). Analysis of Dentinal Stress Distribution of Maxillary Central Incisors Subjected to Various Post-and-Core Applications. *Operative Dentistry*.

[8] Fernandes A. S., Shetty S., Coutinho I. (2003). Factors Determining Post Selection: A Literature Review. *The Journal of Prosthetic Dentistry*.

[9] Bosso K., Gonini Junior A., Guiraldo R. D., Berger S. B., Lopes M. B. (2015). Stress Generated by Customized Glass Fiber Posts and Other Types by Photoelastic Analysis. *Brazilian Dental Journal*.

[10] Pupo Y. M., Casacqui E., de Lima P. A., Michél M. D., Bueno A. L., Michelotto A. L. (2017). Morphology of Root Canal Surface: A Reflection on the Process of Cementation of the Composite Relined Glass Fiber Post. *Indian Journal of Dental Research*.

[11] Mainjot A. K., Dupont N. M., Oudkerk J. C., Dewael T. Y., Sadoun M. J. (2016). From Artisanal to CAD-CAM Blocks: State of the Art of Indirect Composites. *Journal of Dental Research*.

[12] Costa T. S., Brandao R. M. R., Farias Vajgel B. C., SoutoMaior J. R. (2024). CAD-CAM Glass Fiber Compared with Conventional Prefabricated Glass Fiber Posts: A Systematic Review. *The Journal of Prosthetic Dentistry*.

[13] Eid R., Juloski J., Ounsi H., Silwaidi M., Ferrari M., Salameh Z. (2019). Fracture Resistance and Failure Pattern of Endodontically Treated Teeth Restored with Computer-Aided Design/ Computer-Aided Manufacturing Post and Cores: A Pilot Study. *The Journal of Contemporary Dental Practice*.

[14] Gutierrez M. A., Guerrero C. A., Baldion P. A. (2022). Efficacy of CAD/CAM Glass Fiber Posts for the Restoration of Endodontically Treated Teeth. *International Journal of Biomaterials*.

[15] King P. A., Setchell D. J., Rees J. S. (2003). Clinical Evaluation of a Carbon Fibre Reinforced Carbon Endodontic Post. *Journal of Oral Rehabilitation*.

[16] Ferrari M., Vichi A., Mannocci F., Mason P. N. (2000). Retrospective Study of the Clinical Performance of Fiber Posts. *American Journal of Dentistry*.

[17] Garbin C. A., Spazzin A. O., Meira-Junior A. D., Loretto S. C., Lyra A. M., Braz R. (2010). Biomechanical Behaviour of a Fractured Maxillary Incisor Restored with Direct Composite Resin Only or with Different Post Systems. *International Endodontic Journal*.

[18] Spazzin A. O., Galafassi D., de Meira-Junior A. D., Braz R., Garbin C. A. (2009). Influence of Post and Resin Cement on Stress Distribution of Maxillary Central Incisors Restored with Direct Resin Composite. *Operative Dentistry*.

[19] Torbjorner A., Fransson B. (2004). A Literature Review on the Prosthetic Treatment of Structurally Compromised Teeth. *International Journal of Prosthodontics*.

[20] Baratieri L. N. (2002). Abordagem Restauradora De Dentes Tratados Endodonticamente: Pinos/núcleos e Restauraçöes Unitárias. *Odontologia Restauradora: Fundamentos e Possibilidades*.

[21] Clavijo V. G. R. Avaliação DA Resistência à Fratura De Raízes Fragilizadas Reabilitadas Por Diferentes técnicas De Construção De núcleos Intra-Radiculares.

[22] Martins L. R. M., Paulillo L. A. M. S., CTPd A., BdCF B., GRd S., Soares C. J. (2011). Restauração Com Pinos Intrarradiculares Anatômicos Em Grandes Destruições Coronárias. *Revista da Associação Paulista de Cirurgiões Dentistas*.

[23] Hattori M., Takemoto S., Yoshinari M., Kawada E., Oda Y. (2010). Durability of Fiber-Post and Resin Core Build-up Systems. *Dental Materials Journal*.

[24] Batson E. R., Cooper L. F., Duqum I., Mendonca G. (2014). Clinical Outcomes of Three Different Crown Systems with CAD/CAM Technology. *The Journal of Prosthetic Dentistry*.

[25] Sequeira-Byron P., Fedorowicz Z., Carter B., Nasser M., Alrowaili E. F. (2015). Single Crowns versus Conventional Fillings for the Restoration of Root-Filled Teeth. *Cochrane Database of Systematic Reviews*.

[26] Alghazzawi T. F. (2016). Advancements in CAD/CAM Technology: Options for Practical Implementation. *Journal of Prosthodontic Research*.

[27] Toparli M. (2003). Stress Analysis in a Post-Restored Tooth Utilizing the Finite Element Method. *Journal of Oral Rehabilitation*.

[28] Barjau-Escribano A., Sancho-Bru J. L., Forner-Navarro L., Rodriguez-Cervantes P. J., Perez-Gonzalez A., Sanchez-Marin F. T. (2006). Influence of Prefabricated Post Material on Restored Teeth: Fracture Strength and Stress Distribution. *Operative Dentistry*.

[29] Eskitascioglu G., Belli S., Kalkan M. (2002). Evaluation of Two Post Core Systems Using Two Different Methods (fracture Strength Test and a Finite Elemental Stress Analysis). *Journal of Endodontics*.

[30] Holmes D. C., Diaz-Arnold A. M., Leary J. M. (1996). Influence of Post Dimension on Stress Distribution in Dentin. *Journal of Prosthetic Dentistry*.

[31] Sorrentino R., Aversa R., Ferro V. (2007). Three-Dimensional Finite Element Analysis of Strain and Stress Distributions in Endodontically Treated Maxillary Central Incisors Restored with Different Post, Core and Crown Materials. *Dental Materials*.

[32] Reinhardt R. A., Krejci R. F., Pao Y. C., Stannard J. G. (1983). Dentin Stresses in Post-Reconstructed Teeth with Diminishing Bone Support. *Journal of Dental Research*.

[33] Gama M., Balbinot G. S., Ferreira G. C., Mota E. G., Leitune V., Collares F. M. (2021). CAD/CAM Milled Glass Fiber Posts: Adaptation and Mechanical Behavior in Flared Root Canals. *Operative Dentistry*.

[34] Batista V. E. S., Bitencourt S. B., Bastos N. A., Pellizzer E. P., Goiato M. C., Dos Santos D. M. (2020). Influence of the Ferrule Effect on the Failure of Fiber-Reinforced Composite Post-and-Core Restorations: A Systematic Review and Meta-Analysis. *The Journal of Prosthetic Dentistry*.

[35] Bittner N., Hill T., Randi A. (2010). Evaluation of a One-Piece Milled Zirconia Post and Core with Different Post-and-Core Systems: An in Vitro Study. *The Journal of Prosthetic Dentistry*.

[36] Standardization IOf (2019). ISO 4049. Dentistry—Polymer-Based Restorative Materials.

[37] Lopes M. B., Serralvo A. D., Felizardo K. R. (2014). Evaluation of the Flexural Resistance and Stress Contraction of a Silorane-Based Composite Submitted to Different Protocols of Polymerization. *Appli.e.d Adhesion Science*.

[38] Spitznagel F. A., Boldt J., Gierthmuehlen P. C. (2018). CAD/CAM Ceramic Restorative Materials for Natural Teeth. *Journal of Dental Research*.

[39] Moustapha G., AlShwaimi E., Silwadi M., Ounsi H., Ferrari M., Salameh Z. (2019). Marginal and Internal Fit of CAD/CAM Fiber Post and Cores. *International Journal of Computerized Dentistry*.

[40] Falcao Spina D. R., da Costa R. G., Correr G. M., Rached R. N. (2018). Scanning of Root Canal Impression for the Fabrication of a Resin CAD-CAM-Customized Post-and-Core. *The Journal of Prosthetic Dentistry*.

[41] Ruschel G. H., Gomes E. A., Silva-Sousa Y. T. (2018). Mechanical Properties and Superficial Characterization of a Milled CAD-CAM Glass Fiber Post. *Journal of the Mechanical Behavior of Biomedical Materials*.

[42] da Costa R. G., Freire A., de Morais EC C., de Souza E., Correr G. M., Rached R. N. (2017). Effect of CAD/CAM Glass Fiber Post-Core on Cement Micromorphology and Fracture Resistance of Endodontically Treated Roots. *American Journal of Dentistry*.

[43] Bergamo E. T. P., Lopes A. C. O., Campos T. M. B. (2022). Probability of Survival and Failure Mode of Endodontically Treated Incisors without Ferrule Restored with CAD/CAM Fiber-Reinforced Composite (FRC) Post-Cores. *Journal of the Mechanical Behavior of Biomedical Materials*.

[44] Lamichhane A., Xu C., Zhang F. Q. (2014). Dental Fiber-Post Resin Base Material: A Review. *The Journal of Advanced Prosthodontics*.

[45] Basaran E. G., Ayna E., Vallittu P. K., Lassila L. V. (2013). Load Bearing Capacity of Fiber-Reinforced and Unreinforced Composite Resin CAD/CAM-Fabricated Fixed Dental Prostheses. *The Journal of Prosthetic Dentistry*.

[46] Grandini S., Chieffi N., Cagidiaco M. C., Goracci C., Ferrari M. (2008). Fatigue Resistance and Structural Integrity of Different Types of Fiber Posts. *Dental Materials Journal*.

[47] Suzaki N., Yamaguchi S., Nambu E., Tanaka R., Imazato S., Hayashi M. (2021). Fabricated CAD/CAM Post-Core Using Glass Fiber-Reinforced Resin Shows Innovative Potential in Restoring Pulpless Teeth. *Materials (Basel)*.

[48] Bacchi A., Caldas R. A., Schmidt D. (2019). Fracture Strength and Stress Distribution in Premolars Restored with Cast Post-and-Cores or Glass-Fiber Posts Considering the Influence of Ferule. *Biomed Research International*.

[49] Passaretti A., Petroni G., Miracolo G., Savoia V., Perpetuini A., Cicconetti A. (2018). Metal Free, Full Arch, Fixed Prosthesis for Edentulous Mandible Rehabilitation on Four Implants. *Journal of Prosthodontic Research*.

[50] Altitinchi A., Hussein A., Saemundsson S., Clark W., Culp L., Sulaiman T. A. (2024). Anatomic CAD-CAM Post-and-Core Systems: A Mastication Simulation Study. *The Journal of Prosthetic Dentistry*.

